# Early development in children with moderate acute malnutrition: A cross‐sectional study in Burkina Faso

**DOI:** 10.1111/mcn.12928

**Published:** 2019-12-11

**Authors:** Mette F. Olsen, Ann‐Sophie Iuel‐Brockdorff, Charles W. Yaméogo, Bernardette Cichon, Christian Fabiansen, Suzanne Filteau, Kevin Phelan, Albertine Ouédraogo, Jonathan C. Wells, André Briend, Kim F. Michaelsen, Lotte Lauritzen, Christian Ritz, Per Ashorn, Vibeke B. Christensen, Melissa Gladstone, Henrik Friis

**Affiliations:** ^1^ Department of Nutrition, Exercise and Sports, SCIENCE University of Copenhagen Copenhagen Denmark; ^2^ Département Biomédical et Santé Publique Institut de Recherche en Sciences de la Santé Ouagadougou Burkina Faso; ^3^ Faculty of Epidemiology and Population Health London School of Hygiene and Tropical Medicine London UK; ^4^ The Alliance for International Medical Action (ALIMA) Paris France; ^5^ Childhood Nutrition Research Centre UCL Great Ormond Street Institute of Child Health London UK; ^6^ Centre for Child Health Research University of Tampere School of Medicine and Tampere University Hospital Tampere Finland; ^7^ Department of Pediatrics and Adolescent Health Rigshospitalet Copenhagen Denmark; ^8^ Medicins Sans Frontieres – Denmark Copenhagen Denmark; ^9^ Department of Women and Children's Health Institute of Translational Medicine, University of Liverpool Liverpool UK

**Keywords:** Africa, anthropometry, body composition, child development, haemoglobin, moderate acute malnutrition, polyunsaturated fatty acids

## Abstract

Malnutrition impairs cognitive, communication, and motor development, but it is not known how nutrition and health are associated with development in children with moderate acute malnutrition (MAM). We aimed to describe motor and language development of children with MAM and explore its nutrition and health‐related correlates. This cross‐sectional study used baseline data from a nutritional trial in children with MAM aged 6–23 months in Burkina Faso. Motor and language skills were assessed using the Malawi Development Assessment Tool (MDAT). Linear mixed models were used to explore potential correlates of MDAT including socio‐economic status, anthropometry, body composition, whole‐blood polyunsaturated fatty acids (PUFA), haemoglobin (Hb), iron status, and morbidity. We also assessed child and caregiver participation during MDAT procedures and their associations with correlates and development. MDAT data were available for 1.608 children. Mean (95% CI) MDAT *z*‐scores were −0.39 (−0.45, −0.34) for gross motor, 0.54 (0.48, 0.59) for fine motor, and −0.91 (−0.96, −0.86) for language skills. Children with higher mid‐upper arm circumference, weight‐for‐height, height‐for‐age, fat‐free mass, n‐3 PUFAs, Hb, and iron status had better MDAT *z*‐scores, whereas children with more fat mass index, anaemia, illness, and inflammation had poorer *z*‐scores. In addition, children living in larger households or with an unmarried mother had poorer MDAT *z*‐scores. Associations between morbidity and *z*‐scores were largely explained by children's poorer participation during MDAT assessment. The identified factors associated with child development may inform interventions needed to stimulate development during or after management of MAM.

**Key messages**

- Nutrition and health‐related correlates of child development have not previously been described among children with moderate acute malnutrition (MAM).
- The motor and language development of children diagnosed with MAM by low mid‐upper arm circumference (MUAC) did not differ from those diagnosed by low weight‐for‐height z ‐score, when differences in height‐for‐ age were taken into account.
- Higher MUAC and anthropometric z ‐scores, especially height‐for‐age, were associated with better development.
- In addition, more fat‐free mass, better iron status, higher haemoglobin, and n‐3 LC‐PUFA levels were associated with better development, while higher fat mass index, anaemia, illness, inflammation, living in large households, or having an unmarried mother were associated with poorer development among children with MAM.
- There were no differences in development between boys and girls with MAM, but several correlates were sex specific.

## 1 INTRODUCTION

Over 250 million children in low‐ and middle‐income countries are at risk of not meeting their development potential. The highest prevalence is found in Sub‐Saharan Africa, where 66% of the total population under 5 years is at risk (Lu, Black, & Richter, 2016). Malnutrition in early childhood has been linked with impaired cognitive, language, and motor development (Abessa, Bruckers, Kolsteren, & Granitzer, 2017; Grantham‐McGregor, 1995; Sudfeld et al., 2015; van den Heuvel et al., 2017), and studies have shown that the deficits persist even many years after nutritional recovery (Galler et al., 2012; Galler et al., 2012; Lelijveld et al., 2019; Liu, Raine, Venables, & Mednick, 2004; Waber et al., 2018). However, these studies have mainly focused on children with severe acute malnutrition (SAM) or used now outdated definitions of acute malnutrition. Moderate acute malnutrition (MAM) is estimated to affect more than 33 million children globally (Black et al., 2013). It is associated with a threefold risk of death (Olofin et al., 2013) and has also been associated with impaired child development (Sudfeld et al., 2015). But although cognitive development is addressed in guidelines for inpatient management of SAM (WHO, 1999, 2013), it has not been a focus for children with MAM, who are generally managed in the community. The development status of these children, and the factors associated with it, need better understanding in order to be addressed in community programmes.

MAM is currently defined by two criteria: weight‐for‐height *z* score (WHZ) between −2 and −3 and/or a mid‐upper arm circumference (MUAC) between 115 and 125 mm (WHO, UNICEF, WFP and UNHCR, 2010). Whether WHZ‐ or MUAC‐based criteria are more suitable for identifying children in need of treatment is still under debate (Briend et al., 2016; Grellety & Golden, 2018; Hossain et al., 2017; Tadesse, Tadesse, Berhane, & Ekström, 2017), but children falling under each of the categories have not yet been well characterised, including with regard to their motor and language development. The association of linear and ponderal growth with child development has been reported from several low‐ and middle‐income settings (Adair et al., 2013; Prado et al., 2017; Worku et al., 2018). However, even in individuals with similar anthropometry, body composition may differ considerably (Deurenberg, Yap, & van Staveren, 1998), and children who are acutely malnourished may be at different risk of developmental delay depending on whether relatively more lean or fat mass has been lost. Recently, the importance of fat‐free mass (FFM) for child development was highlighted in an Ethiopian study showing that FFM at birth (Abera et al., 2017) and accretion during early infancy (Abera et al., 2018) predicted developmental status later in childhood. Other factors of potential importance to brain development among children with MAM include long‐chain polyunsaturated fatty acids (LC‐PUFA), haemoglobin (Hb), iron status, morbidity, and socio‐economic characteristics of children (González et al., 2018; Nyaradi, Li, Hickling, Foster, & Oddy, 2013; Prado & Dewey, 2014). The aim of this study was to describe motor and language development of 6–23‐month‐old children with MAM in Burkina Faso and to explore nutrition and health‐related correlates including socio‐economic status, anthropometry, MAM‐defining criteria, body composition, LC‐PUFA, Hb, iron status, illness, and inflammation.

## 2 METHODS

### 2.1 Study design and participants

This is a cross‐sectional study using baseline data collected between September 2013 and August 2014 as part of the Treatfood study, a randomised controlled trial testing effectiveness of food supplements for young children with MAM. The study's primary outcome was increase of FFM index, whereas child development was included as a secondary outcome (trial registration: ISRCTN42569496). As previously described (Fabiansen et al., 2017), the study was carried out in five health centres in the Province du Passoré, Burkina Faso, with a catchment area covering a total of 143 villages and a total population of approximately 258,000. Study inclusion criteria were residency within catchment area, confirmed MAM diagnosis (MUAC ≥115 mm and <125 mm and/or WHZ ≥−3 and <−2), and age 6–23 months. Children were excluded if they were diagnosed with SAM (WHZ <−3 or MUAC <115 mm or oedema), already in a nutrition programme, required hospitalisation, or hospitalised within the past 2 months. Children with overt disability, limiting the feasibility of investigations, or with suspected allergies to ingredients in the tested supplements were also excluded. Caregivers gave verbal and written consent prior to enrolment. The study was approved by the Ethics Committee for Health Research in Burkina Faso (2012‐8‐059), and consultative approval was obtained from the Danish National Committee on Biomedical Research Ethics (1208204).

### 2.2 Assessment of child development

Child development was assessed using an adapted version of the Malawi Development Assessment Tool (MDAT; Gladstone et al., 2010). The tool was pilot tested in the setting by one of the investigators (A‐S I‐B), who received training in Malawi on its use. The original MDAT is intended for assessing development of children ≤5 years in four domains of each 34 items (gross motor, fine motor, language, and social). As the Treatfood study involved long study visits with many other activities, including collection of blood and saliva samples, we decided to only include three domains (gross motor, fine motor, and language) to reduce the risk of children lacking concentration for the MDAT activities. Also, only the first 30 items of each domain were included due to the young age of participants. Items were rated as passed (1 point) or failed (0 points). After having failed six consecutive items within a domain, the assessor would mark the remaining items as failed and move to the next domain.

Adaption and validation of the MDAT was undertaken in collaboration with two local research assistants with degrees in sociology. All items were found to be culturally relevant, although some naming objects were replaced with objects more familiar in the setting (i.e., a doll, a car, and a glass with screw lid were replaced by sandals, clothing, and a cola bottle). The MDAT was translated into French and semantic equivalence ensured using back translation. Because the local language of Moré is not commonly used as a written language, the research assistants preferred to have the tool in French and translate verbally during evaluation. The verbal translations of each item in Moré were carefully discussed between the research assistants to ensure semantic and conceptual equivalence. Three informal focus group discussions were carried out with a total of 18 mothers, and on the basis of these, we concluded that MDAT sessions and items were appropriate and well accepted in the setting.

To assess interrater and intrarater reliability of the MDAT, we conducted a pilot study including a convenience sample of a total of 56 children aged 6–30 months of whom 33 (59%) were girls, 19 (34%) had MAM, and five (9%) had SAM. Three MDAT assessors were involved in interrater reliability tests, which were based on duplicate evaluations of 34 children. Video recordings were used in subsequent discussions when evaluations disagreed. Intraclass correlation coefficients based on linear mixed models showed high agreement for all domains (gross motor: 0.97, fine motor: 0.96, and language: 0.90). Intra‐rater reliability was assessed based on repeated evaluations of 11 children 2 days apart. Intraclass correlation coefficients showed very high or high agreement (gross motor: 0.96, fine motor: 0.88, and language: 0.73).

When assessing child development, it is a challenge to differentiate between children's performance on the day of evaluation and their underlying development status. The assessors therefore evaluated participation during MDAT assessment using five items rating the mood, engagement, cooperativeness and shyness of the child, and the involvement of the caregiver. These items were adapted from the Behaviour Observation Inventory from the Bayley Scales of Infant and Toddler Development (Bayley, 2005; Tofail et al., 2013) and have previously been used to supplement MDAT assessments (van den Heuvel et al., 2017). In duplicate evaluations of 34 children in the pilot study, the interrater reliability of child participation was poor to moderate (kappa correlation coefficients: 0.10–0.57) and poor to fair for maternal encouragement (kappa correlation coefficients: 0.07–0.35). Acknowledging that MDAT data reflect both children's abilities and willingness to participate in the tasks, we chose to include the participation observations despite their low interrater reliability.

### 2.3 Sociodemographic and clinical data collection

At enrolment, a research nurse collected data on sociodemographic characteristics (including age, sex, ethnicity, religion, parental education, occupation and marital status, fuel used for cooking, house ownership, and household size defined as number of people sharing family meals), current breastfeeding status, and 14‐day retrospective history of morbidity (including illness and fever) using a structured questionnaire. The nurse also diagnosed diarrhoea and infections based on an adapted version of the Integrated Management of Childhood Illnesses guideline (World Health Organization, 2005).

### 2.4 Anthropometric measures

Weight was measured in duplicate to the nearest 100 g using electronic scales (Seca model 881 1021659). Length was measured in duplicate to the nearest 1 mm with a wooden height board. The STATA package “zscore06” was used to calculate WHZ and height‐for‐age *z*‐scores (HAZ; WHO Multicentre Growth Reference Study Group, 2006). MUAC was measured in duplicate to the nearest 1 mm, at the midpoint between the olecranon and the acromion process of the left arm using a standard measuring tape.

### 2.5 Body composition

The deuterium dilution technique was used for assessment of total body water. FFM was calculated as total body water/hydration, using age‐ and sex‐specific hydration coefficients (Lohman, 1992), and fat mass (FM) was calculated as weight minus FFM. FFM and FM were divided with length in meters squared to derive length‐adjusted fat‐free mass index (FFMI) and fat mass index (FMI; VanItallie, Yang, Heymsfield, Funk, & Boileau, 1990). Details of the deuterium dilution technique and its local adaptation have been described elsewhere (Fabiansen et al., 2017; Fabiansen, Yaméogo, Iuel‐Brockdorf, et al., 2017).

### 2.6 Blood sampling and analysis

A 2.5 mL of venous blood was collected from the arm and used to saturate 1 cm^2^ of a chromatography paper strip treated with 50‐μg 2,6‐di‐tert‐butyl‐4‐methylphenol (butylated hydroxytoluene) and 1,000‐μg deferoxamine mesylate salt (both from Sigma‐Aldrich, St. Louis, MI, USA) for analysis of whole‐blood fatty acid composition, as previously described (Yaméogo et al., 2017). Data are given as percent by weight of individual fatty acids relative to the total fatty acid concentration in each sample (FA%). We regarded the triene to tetraene ratio (defined as Mead acid (C20:3n‐9):AA (arachidonic acid, C20:4n‐6) as an indicator of low overall PUFA status (Sardesai, 1992), whereas the n‐6 DPA (docosapentaenoic acid, C22:5n‐6):DHA (docosahexaenoic acid, C20:6n‐3) and n‐6:n‐3 PUFA ratios were regarded as indicators of low n‐3 PUFA status as in previous reports from the study (Yaméogo et al., 2017).

Hb was measured on site using a HemoCue device (Hb 301, Ängelholm). The remaining blood was put into a sample tube with clot activator (BD reference #368492) and transported to the trial laboratory in a cold box at 2–8°C. Serum was isolated after centrifugation at 700 × *g* for 5 min (EBA 20 S; Hettich) at room temperature and stored at −20^o^C until shipment to the VitMin Lab in Willstaedt, Germany, for analysis of C‐reactive protein (CRP), serum ferritin (SF), and soluble transferrin receptors (sTfR) with the use of a combined sandwich ELISA (Erhardt, Estes, Pfeiffer, Biesalski, & Craft, 2004). In addition, α_1_‐acid glycoprotein (AGP) was analysed for use in SF adjustment. All samples were analysed in duplicate, and both intraassay and interassay CVs were <10%. Samples were frozen and thawed only once before analysis. As previously described, SF data were corrected for inflammation using a linear model with CRP, AGP, and morbidity (malaria, lower respiratory tract infections, and history of fever; Cichon et al., 2017). Malaria (*Plasmodium falciparum*) was diagnosed from venous blood using a rapid diagnostic test (SD Bioline Malaria Ag Pf).

### 2.7 Data handling and statistical analysis

Data were double‐entered in EpiData 3.1 (EpiData Association, Odense, Denmark). Statistical analyses were carried out using STATA 14.2 (StataCorp, College Station, TX, USA). Descriptive data are shown as mean ±standard deviation (SD), median (interquartile range), or number (%).

We calculated MDAT *z*‐scores based on reference data from a Malawian nonmalnourished population (Gladstone et al., 2010). As reference data contained 34 items in each domain, we imputed four items in our data. The majority of children (94.5%) had already failed six consecutive items during their assessment, and the additional items were thus marked as “failed.” The remaining children were given the mean of their last six‐item scores in the imputed items. We conducted a sensitivity analysis, where these children were given minimum and maximum values, respectively, to check if imputations could have an impact on analyses. Domain *z*‐scores below −1.64 were considered suspect for developmental delay (Gladstone et al., 2010).

Linear mixed models were used to explore correlates of fine motor, gross motor, and language *z*‐scores, respectively. The potential correlates included sociodemographic characteristics (sex, parental education level and marital status, household size, house ownership, and fuel used for cooking), anthropometry (MUAC, WHZ, HAZ, and MAM‐defining criteria), body composition (fat and FFM), LC‐PUFA status (DHA, AA, Mead acid: AA ratio, and n‐6 DPA:DHA ratio included in main analyses, additional analyses in the Supporting Information), Hb and iron (Hb, inflammation‐corrected SF, sTfR, and anaemia), and morbidity (illness within last 2 weeks, malaria, and serum CRP). We assessed the role of MAM‐defining criteria by comparing three groups of children: (a) low MUAC only, (b) low WHZ only, and (c) low MUAC and low WHZ. In the assessment of WHZ as a potential correlate of MDAT scores, we included all children with MAM defined by the WHZ criteria. Similarly, the assessment of MUAC as a correlate of MDAT scores was done among the children with MUAC below the MAM‐defining criteria. All models included adjustment for age, sex, month of inclusion, and site‐specific random effects. For selected correlates (MAM‐defining criteria and body composition), we also considered models including further adjustment for HAZ to check for confounding. We tested if sex acted as an effect modifier for any of the potential correlates. Last, we explored the role of children's performance of the day of evaluation by assessing associations between child and maternal participation during MDAT evaluations, health and nutrition indicators, and MDAT *z*‐scores, respectively. Associations with *P* values <.05 were considered significant.

### 2.8 Ethical statement

Caregivers gave verbal and written consent prior to enrolment. The trial was approved by the Ethics Committee for Health Research in Burkina Faso (2012‐8‐059), and consultative approval was obtained from the Danish National Committee on Biomedical Research Ethics (1208204). The trial was registered in the ISRCTN registry (ISRCTN42569496).

## 3 RESULTS

MDAT data were available for 1,608 of the 1,609 children recruited for the Treatfood trial. Children had a median age of 11.3 months, and 55% were girls (Table 1). Most parents had no formal schooling (86% of mothers, 83% of fathers). The majority of parents worked in agriculture (93%), and other occupations included trade, animal husbandry, manual labour, and gold panning. The main ethnic group was Mossi (94%), and the main religion of mothers was Islam (59%) followed by Catholicism (24%) and traditional animism (11%). Polygamy was common in the setting, and several generations often lived together, resulting in large households ranging from 3 to 80 members. There was very little variation in other socio‐economic characteristics (e.g., 99% lived in a house owned by one of the household members and 99% used coal as cooking fuel). Data on anthropometry, MAM‐defining criteria, body composition, LC‐PUFA, Hb, iron status, and morbidity are presented in Table 1 for information and described in more detail in previous publications (Cichon et al., 2016; Fabiansen, Yaméogo, Iuel‐Brockdorf, et al., 2017; Yaméogo et al., 2017); 27% of children were moderately stunted (HAZ between −2 and −3) and 10% severely stunted (HAZ <−3). Morbidity was very common with 38% recently ill and 40% having a positive malaria test on the day of MDAT assessment.

**Table 1:** Characteristics of 1,608 children with moderate acute malnutrition

	*n*	
Sociodemographic characteristics		
Age (months)	1,608	11.3 [8.2–16.0]
Sex (girls)	1,608	879 (54.7)
Maternal education level	1,602	
None		1,373 (85.7)
Primary school incomplete		127 (7.9)
Primary school complete or higher		102 (6.4)
Maternal marital status	1,597	
Married, monogamous		645 (40.4)
Married, polygamous		696 (43.6)
Unmarried		256 (16.0)
Household size, median [IQR], (range)	1,608	10 [7‐16], (3–80)
Breastfeeding		
Child still breastfed	1,608	1,520 (94.7)
Anthropometry		
Mid‐upper arm circumference (MUAC), mm	1,608	122.6 ±4.0
Weight‐for‐height *z*‐score (WHZ)	1,608	−2.22 ±0.51
Height‐for‐age *z*‐score (HAZ)	1,608	−1.70 ±1.12
Admission criteria	1,608	
Mid‐upper arm circumference (MUAC) only		467 (29.0)
Weight‐for‐height *z*‐score (WHZ) only		337 (21.0)
WHZ and MUAC		804 (50.0)
Body composition		
Fat‐free mass, kg	1,489	5.79 ± 0.91
Fat mass, kg	1,489	1.13 ± 0.39
Fat‐free mass index, kg/m^2^	1,489	11.62 ± 0.87
Fat mass index, kg/m^2^	1,489	2.30 ± 0.78
Whole blood polyunsaturated fatty acids (PUFA)^a^		
n‐3 PUFA, FA%	1,572	2.48 ±0.66
n‐6 PUFA, FA%	1,572	26.00 ±2.66
Docosahexaenoic acid (DHA, C22:6n‐3), FA%	1,572	1.64 ±0.53
Arachidonic acid (AA, C20:4n‐6), FA%	1,572	7.08 ±1.54
Indicator of low PUFA status		
Mead acid: AA ratio	1,572	0.01 ±0.005
Indicators of low n‐3 PUFA status		
n‐6 docosapentaenoic acid (C22:5n‐6):DHA ratio	1,572	0.25 ±0.10
n‐6 PUFA:n‐3 PUFA ratio	1,572	11.13 ±2.86
Haemoglobin and iron		
Haemoglobin, g/dL	1,608	10.02 ±1.59
Serum ferritin (SF), μg/L	1,564	33.4 [13.5–74.0]
SF corrected for inflammation^b^, μg/L	1,555	16.0 [8.0–30.0]
Soluble transferrin receptors, mg/L	1.564	12.6 [9.1–17.3]
Anaemia with iron deficiency^c^	1.555	469 (30.2)
Anaemia without iron deficiency ^d^	1.555	618 (39.7)
Morbidity		
Illness within the last two weeks	1,599	608 (38.0)
Malaria (positive test)	1,600	644 (40.3)
C‐reactive protein, mg/L	1,564	2.3 [0.8–9.4]

*Note*. Data are mean ±SD, median [IQR], or n (%).

a LCPUFA data are given in weight percent relative to total fatty acid concentration (FA%). Mean fatty acid concentration was 417 (±183) μg/100 μl whole blood (full list of fatty acids available in the Supporting Information).

b Ferritin data corrected in linear model with C‐reactive protein (CRP), α_1_‐acid glycoprotein (AGP), and morbidity covariates (malaria, lower respiratory tract infections, and history of fever).

c Defined as haemoglobin <11 g/dl and SFAI <12 μg/L.

Defined as haemoglobin <11 g/dl and SFAI ≥12 μg/L.

MDAT *z*‐scores and the proportion with suspected delay showed that developmental deficits were largest in the language domain (Table 2). Linear mixed models with adjustment for age, sex, month of inclusion, and site (random effects) showed that boys and girls had similar MDAT *z*‐scores in all domains (Table 3). Living in larger households or with unmarried mothers was associated with poorer development. We did not find an association between maternal education, and MDAT *z*‐scores and additional socio‐economic characteristics were not assessed due to the limited variation described above. All anthropometric indicators were associated with development scores. Having a higher MUAC was associated with better gross and fine motor *z*‐scores (both 0.12 *z* score per 1 SD increase of MUAC), whereas higher WHZ was only associated with better fine motor scores (0.19 *z* score per 1 SD increase of WHZ). Among the anthropometric indicators, HAZ was the strongest correlate with coefficients of 0.32 motor development *z*‐score per 1 SD increase of HAZ. In the language domain, the coefficient of HAZ was markedly higher for boys than girls (0.29 vs. 0.13 *z* score per 1 SD increase of HAZ, test of interaction: *P* = .002). When comparing the MAM‐defining criteria, children recruited based on low MUAC apparently had lower *z*‐scores in all development domains than children with low WHZ. However, this was confounded by differences in children's height, because children with low MUAC were shorter‐for‐age than children with low WHZ. Thus, their MDAT *z*‐scores were similar in the HAZ‐adjusted analyses.

**Table 2:** MDAT *z*‐scores and suspected delay in 1.608 children with moderate acute malnutrition

MDAT domain	Mean *z*‐score (95% CI)	Developmental delay, *n* (%)
Gross motor	−0.39 (−0.45, −0.34)	186 (11.6)
Fine motor	0.54 (0.48, 0.59)	74 (4.6)
Language	−0.91 (−0.96, −0.86)	390 (24.3)

*Note*. MDAT = adapted version of the Malawi Development Assessment Tool. *Z*‐scores are based on MDAT reference population data (Gladstone 2010). Domain *z*‐scores below −1.64 are suspect for developmental delay.

**Table 3:** Sociodemographic and anthropometric correlates of MDAT z‐scores in 1.608 children with moderate acute malnutrition

		Gross motor domain		Fine motor domain		Language domain	
	*n*	*β* (95% CI)	*p*	*β* (95% CI)	*P*	*β* (95% CI)	*P*
Sociodemographic characteristics							
Sex							
Boys	729	Ref		Ref		Ref	
Girls	879	−0.06 (−0.16, 0.04)	.22	−0.04 (−0.14, 0.06)	.43	0.03 (−0.06, 0.12)	.53
Maternal education level							
None	1,373	Ref	.63	Ref	.90	Ref	.87
Primary school incomplete	156	0.09 (−0.09, 0.28)		0.02 (−0.17, 0.21)		−0.03 (−0.20, 0.14)	
Primary school complete or higher	73	0.01 (−0.19, 0.22)		0.04 (−0.17, 0.25)		0.04 (−0.15, 0.22)	
Maternal marital status							
Married, monogamous	645	Ref		Ref		Ref	
Married, polygamous	696	−0.10 (−0.21, 0.01)	.07	−0.03 (−0.14, 0.08)	.56	−0.07 (−0.17, 0.03)	.17
Unmarried	256	−0.20 (−0.35, −0.05)	.01	−0.05 (−0.21, 0.10)	.49	−0.01 (−0.15, 0.13)	.89
Household size							
≤6 household members	263	Ref	.01	Ref	.10	Ref	.02
7–12 household members	713	−0.02 (−0.16, 0.13)		−0.02 (−0.16, 0.13)		−0.04 (−0.17, 0.09)	
≥13 household members	632	−0.17 (−0.32, −0.02)		−0.13 (−0.28, 0.02)		−0.17 (−0.30, −0.03)	
Anthropometry							
Mid‐upper arm circumference (MUAC), mm^a^	1,271	0.03 (0.01, 0.05)	.002	0.03 (0.01, 0.05)	.01	−0.004 (−0.02, 0.02)	.67
Weight‐for‐height z‐score (WHZ)^a^	1,141	0.07 (−0.13, 0.29)	.47	0.37 (0.17, 0.58)	<.001	0.16 (−0.03, 0.36)	.10
Height‐for‐age z‐score (HAZ)	1,608Boys: 729Girls: 879	0.29 (0.24, 0.33)	<.001	0.29 (0.24, 0.33)	<.001	Interaction: *P* = .002^c^ 0.26 (0.20, 0.32)0.12 (0.06, 0.18)	<.001<.001
MAM‐defining criteria							
MUAC only	467	Ref		Ref		Ref	
WHZ only	337	0.31 (0.15, 0.47)	<.001	0.24 (0.08, 0.40)	.004	0.16 (0.02, 0.30)	.03
MUAC and WHZ	804	0.06 (−0.06, 0.18)	.32	0.10 (−0.02, 0.22)	.11	0.05 (−0.06, 0.16)	.37
WHZ only, adjusted for HAZ	337	0.06 (−0.09, 0.22)	.41	−0.01 (−0.17, 0.15)	.89	−0.01 (−0.15, 0.14)	.91
MUAC and WHZ, adjusted for HAZ	804	−0.04 (−0.16, 0.08)	.49	−0.004 (−0.12, 0.11)	.94	−0.02 (−0.13, 0.09)	.71
Body composition							
Fat‐free mass, kg	1,489	0.48 (0.39, 0.58)	<.001	0.38 (0.29, 0.48)	<.001	0.30 (0.21, 0.39)	<.001
Fat mass, kg	1,489	−0.03 (−0.17, 0.12)	.72	0.14 (−0.003, 0.28)	.056	−0.04 (−0.17, 0.09)	.55
Fat‐free mass index, kg/m^2^	1,489	0.02 (−0.05, 0.08)	.58	−0.03 (−0.09, 0.04)	.39	0.01 (−0.05, 0.07)	.73
Fat mass index, kg/m^2^	1,489	−0.13 (−0.20, −0.05)	.001	−0.03 (−0.11, 0.04)	.37	−0.07 (−0.14, −0.05)	.036
Fat‐free mass index, kg/m^2^, adjusted for HAZ	1,489	0.10 (0.04, 0.16)	.002	0.05 (−0.01, 0.11)	.12	0.07 (0.01, 0.12)	.03
Fat mass index, kg/m^2^, adjusted for HAZ	1,489	−0.11 (−0.18, −0.04)	.002	−0.02 (−0.09, 0.05)	.61	−0.06 (−0.13, 0.005)	.07

*Note*. Data are mean difference (95% CI) from linear mixed models adjusted for age, sex, month of inclusion, and site (random effects).

Includes children with MUAC <125 mm only.

a Includes children with WHZ <−2 only.

Due to interaction, sex‐specific estimates are given.

When assessing body composition, we found that FFM was a very strong correlate of motor and language development (e.g., 0.44 gross motor *z*‐score per 1 SD increase in FFM), whereas FM was only marginally associated with better fine motor *z*‐scores. However, when body composition was expressed as indices (kg/m^2^), FFMI was not associated with MDAT *z*‐scores, whereas higher FMI was associated with poorer gross motor and language *z*‐scores (−0.10 gross motor *z*‐score per 1 SD increase in FMI). In models of the indices with further adjustment for HAZ, FFMI was associated with better, whereas FMI was associated with worse gross motor and language *z*‐scores.

Selected indicators of fatty acid levels are presented in Table 4, and a full presentation is given in Table S1. In brief, these data show that children with higher total PUFA levels had better *z*‐scores in all MDAT domains and that this was primarily driven by n‐3 LC‐PUFAs, whereas n‐6 LC‐PUFAs were mainly associated with better gross motor *z*‐scores. One of the strongest LC‐PUFA correlates was DHA with 0.15 increase in gross motor *z*‐score per 1 SD increase in DHA. The indicators of low n‐3 PUFA status were associated with poorer *z*‐scores in all MDAT domains (e.g., −0.11 gross motor *z*‐score per 1 SD increase of the n‐6:n‐3 PUFA ratio). Several of the PUFA correlates were sex specific: Higher α‐linoleic acid (ALA) was associated with poorer gross motor *z*‐scores among boys only (interaction: *P* = .02), whereas higher n‐6 DPA was associated with poorer language skills and a higher n‐6 DPA:DHA ratio with poorer fine motor skills among girls only (interaction: *P* = .008 and *P* = .04, respectively).

**Table 4:** Biochemical and clinical correlates of MDAT *z*‐scores in 1.608 children with moderate acute malnutrition

		Gross motor domain		Fine motor domain		Language domain	
	*n*	*β* (95% CI)	*P*	*β* (95% CI)	*P*	*β* (95% CI)	*P*
Long‐chain polyunsaturated fatty acids, %FA^a^							
Docosahexaenoic acid (DHA)	1,572	0.28 (0.18, 0.38)	<.001	0.11 (0.01, 0.21)	.04	0.15 (0.06, 0.24)	.001
Arachidonic acid (AA)	1,572	0.08 (0.04, 0.11)	<.001	0.01 (−0.02, 0.05)	.45	0.02 (−0.01, 0.05)	.22
Indicator of low PUFA status							
Mead acid: AA ratio	1,572	−10.96 (−21.44, −0.49)	.04	−1.99 (−12.61, 8.63)	0.71	3.88 (−5.66, 13.41)	.43
Indicator of low n‐3 PUFA status							
n‐6 docosapentaenoic acid (n‐6 DPA): DHA ratio	1,572Boys: 710Girls: 862	−0.84 (−1.37, −0.32)	.002	Interaction: p=0.04^b^−0.25 (−1.02, 0.52)−1.32 (−2.07, −0.58)	.53<.001	−0.62 (−1.10, −0.14)	.01
Haemoglobin and iron							
Hb, g/dL	1,608	0.11 (0.07, 0.14)	<.001	0.08 (0.04, 0.11)	<.001	0.06 (0.03, 0.09)	<.001
Serum ferritin, inflammation‐corrected^b^, ln (μg/L)	1,555Boys: 700Girls: 855	−0.01 (−0.06, 0.05)	.86	0.001 (−0.06, 0.06)	.97	Interaction: *P* = .045^b^ 0.08 (0.004, 0.16)−0.02 (−0.09, 0.04)	.04.49
≥12 μg/L	595	Ref		Ref		Ref	
<12 μg/L	960	−0.004 (−0.11, 0.10)	.94	0.04 (−0.07, 0.15)	.45	−0.04 (−0.14, 0.06)	.42
Soluble transferrin receptors, ln (mg/L)	1,564	−0.36 (−0.48, −0.24)	<.001	−0.17 (−0.30, −0.05)	.006	−0.21 (−0.32, −0.10)	<.001
≤8.3 mg/L	268	Ref	<.001	Ref	.03	Ref	.02
>8.3–<15 mg/L	742	−0.08 (−0.22, 0.07)		−0.001 (−0.15, 0.14)		0.003 (−0.13, 0.13)	
≥15 mg/L	554	−0.34 (−0.50, −0.19)		−0.15 (−0.31, 0.01)		−0.14 (−0.28, 0.002)	
Anaemia							
No anaemia	468	Ref		Ref		Ref	
Anaemia with iron deficiency c	469	−0.19 (−0.32, −0.05)	.006	−0.15 (−0.28, −0.01)	.04	−0.13 (−0.25, −0.01)	.04
Anaemia without iron deficiency ^e^	618	−0.16 (−0.28, −0.03)	.02	−0.19 (−0.32, −0.06)	.004	−0.11 (−0.23, 0.001)	.051
Morbidity							
Illness within the last two weeks							
No	991	Ref		Ref		Ref	
Yes	608	−0.17 (−0.27, −0.06)	.003	−0.20 (−0.31, −0.09)	<.001	−0.13 (−0.23, −0.03)	.01
Malaria (positive test)							
Negative test	956	Ref		Ref		Ref	
Positive test	644	−0.14 (−0.25, −0.02)	.02	−0.10 (−0.22, 0.02)	.10	−0.06 (−0.17, 0.04)	.25
Serum CRP, ln (mg/L)	1,555	−0.06 (−0.09, −0.03)	<.001	−0.08 (−0.11, −0.05)	<.001	−0.07 (−0.09, −0.04)	<.001
<5 mg/L	1,002	Ref	<.001	Ref	<.001	Ref	<.001
≥5 to <10 mg/L	183	−0.19 (−0.35, −0.03)		−0.10 (−0.27, 0.06)		−0.11 (−0.26, 0.04)	
≥10 mg/L	379	−0.21 (−0.33, −0.09)		−0.29 (−0.42, −0.17)		−0.25 (−0.37, −0.14)	

*Note*. Data are mean differences (95% CI) from linear mixed models adjusted for age, sex, month of inclusion, and site (random effects).

a LCPUFA data are given in weight percent relative to total fatty acid concentration (FA%).

Due to interaction, sex‐specific estimates are given.

b Corrected in linear model with C‐reactive protein (CRP), α_1_‐acid glycoprotein (AGP), and morbidity covariates (malaria, lower respiratory tract infections, and history of fever).

c Defined as haemoglobin <11 g/dl and SFAI <12 μg/L.

Defined as haemoglobin <11 g/dL and SFAI ≥12 μg/L.

Both Hb and sTfR were associated with all MDAT domains (e.g., gross motor *z*‐scores were 0.17 higher per 1 SD increase in Hb and 0.16 lower per 1 SD increase in ln (sTfR)). In line with this, children with anaemia had poorer *z*‐scores in all MDAT domains. However, an association between higher inflammation‐corrected levels of SF and better development was only seen for language *z*‐scores among boys (interaction: *P* = .045). Finally, measures of morbidity were associated with poorer MDAT *z*‐scores. Recent illness and inflammation were associated with poorer *z*‐scores in all domains (e.g., 0.06 higher gross motor *z*‐score per 1 SD increase of ln (CRP)), whereas a positive malaria test was associated with poorer gross motor *z*‐scores only. Sensitivity analyses showed that of the estimation of MDAT *z*‐scores, and their correlates was unaffected by imputation of missing items (Tables S2–S4).

During MDAT assessment, most of the children were in a good mood, engaged and enthusiastic about the activities, cooperative with the instructions given, and did not appear shy (Table 5). However, 21% of the children were rated as “not very cooperative” and 11% as “uncooperative.” Children who were less cooperative also had poorer MDAT *z*‐scores and were more likely to have been ill recently, for example, 15% who had been ill were assessed as uncooperative, in contrast to 8% of other children. No differences were observed in participation between boys and girls, but younger children were generally more engaged and cooperative than older children. In addition, higher levels of LC‐PUFA and Hb were associated with better cooperativeness (data not shown). Nearly all children (99%) were accompanied by their mother. About half of them actively encouraged the child during the MDAT assessment, whereas others encouraged more passively or watched without involvement (Table 5). The level of encouragement was associated with higher MDAT *z*‐scores as well as children's age and anthropometry, so that older and larger (higher MUAC/WHZ/HAZ) children received more encouragement than younger and smaller children. No difference was observed in encouragement given to boys and girls (data not shown).

**Table 5:** Evaluation of child and maternal participation during MDAT assessment

	*n* (%)
Child mood during the majority of assessment	
Happy, smiling and laughing	267 (16.6)
Mostly happy	907 (56.4)
Neutral	188 (11.7)
Mostly sad, crying, or complaining	202 (12.6)
Very sad, crying, or complaining	43 (2.7)
Child engagement/enthusiasm with activities	
Interested, engaged, and enthusiastic	317 (19.7)
Mostly interested and engaged	750 (46.7)
A little interested but easily distracted	373 (23.2)
Uninterested and not engaged	167 (10.4)
Child cooperativeness	
Does what assessor asks him/her to do	825 (51.5)
A little slow to cooperat, but does so most of the time.	276 (17.2)
Not very cooperative	329 (20.5)
Very difficult and uncooperative	173 (10.8)
Child shyness/anxiety	
Not shy or nervous	1,042 (65.0)
A little shy or anxious	456 (28.4)
Very anxious or scared	75 (4.7)
Too anxious or scared to engage in activities	31 (1.9)
Maternal encouragement	
Strongly and actively encourages the child	864 (53.9)
Passively encourages without much involvement	510 (31.8)
Watched passively	226 (14.1)
Actively discourages the child	4 (0.3)

Abbreviation: MDAT, Malawi Development Assessment Tool.

Because cooperativeness during MDAT assessment was associated with MDAT *z*‐scores as well as Hb, PUFAs (DHA and AA), and morbidity, we repeated models assessing these correlates with inclusion of the level of cooperativeness. We found that for Hb, DHA, and AA, the adjusted estimates were only moderately attenuated (e.g., from 0.15 to 0.14 gross motor *z*‐score per 1 SD increase in DHA), whereas the associations with recent illness, positive malaria test, and CRP were largely explained by lower cooperativeness (e.g., from −0.17 to −0.10 gross motor *z*‐score among children with recent illness, which was no longer significant, *P* = .07). Similarly, as we found caregivers' encouragement was positively associated with children's size (MUAC/WHZ/HAZ) and MDAT *z*‐scores, we also repeated models of these anthropometric indicators with inclusion of maternal encouragement. However, adjusted estimates were similar to unadjusted for all correlates.

## 4 DISCUSSION

In this study, we have described the status of motor and language development among 6–23‐month‐old children diagnosed with MAM in Burkina Faso and shown how it was associated with their health and nutritional characteristics. We found that children with higher anthropometric *z*‐scores, especially HAZ, had better development scores. Children with MAM diagnosed by the MUAC‐criteria had similar development scores to those diagnosed by the WHZ‐criteria, once differences in HAZ had been taken into account. Body composition was also of importance to child development as FFM was associated with better development scores, whereas FMI was associated with poorer development. In addition, higher Hb, SF (indicating better iron stores), and n‐3 LC‐PUFA levels were associated with better development, whereas higher sTfR (indicating iron deficient tissues), anaemia, illness, and inflammation were associated with poorer development. A relatively homogenous study population limited our ability to assess socio‐economic correlates of development. However, we did find that children living in large households or with an unmarried mother had poorer development scores. There were no differences in MDAT *z*‐scores between boys and girls, but several correlates of MDAT were modified by sex, for example, the association between higher HAZ and better language development was much stronger among boys. Variation in children's cooperativeness during MDAT assessment partly explained associations between morbidity, but not nutritional, correlates and MDAT *z*‐scores, whereas caregivers' encouragement during MDAT assessment did not influence any of the estimated associations.

The main strength of this study was the access to a wide range of clinical and nutritional data from a large trial setting, which allowed a detailed assessment of potential correlates of motor and language development among children with MAM. In addition, we applied a tool for child development assessment, which was validated in the setting with high interrater and intrarater reliability. The main limitation of our study is its cross‐sectional design, which does not allow us to conclude on the directions of associations or pathways of development. We acknowledge that the exploratory nature of analyses and the many potential correlates considered implies a risk of type I errors. As mentioned, the study was also limited by sparse demographic variation of the population, reducing our chances of identifying socio‐economic correlates of development. We lacked information about additional environmental factors, which may have been of importance, such as household assets, stimulation, or adversities in the household. Furthermore, we acknowledge that there was poor interrater reliability of the evaluations of MDAT participation, but despite this, the data allowed us to explore differences between children's engagement in tasks and their actual abilities. Last, there was considerable variability of the MDAT data, but due to the large sample, we were able to detect rather small associations. We acknowledge that some of the coefficient sizes might be too small to be of relevance for the children's development, whereas others were large enough to be of biological importance, for example, an increase of 1 SD HAZ was associated with 0.3 SD increase in motor *z*‐scores.

Although this is the first study to describe child development in a MAM population, our findings concur with a prospective study among 4,205 children in Ghana, Malawi, and Burkina Faso, where identified predictors of motor and language development included linear and ponderal growth, Hb, and iron status (Prado et al., 2017). A strong association between linear growth and development was also seen in this MAM population, where HAZ was the strongest anthropometric correlate of motor and language development. However, linear growth should not be seen as a direct causal factor for child development but rather as an indicator of the adequacy of previous diet, health, and care in the child's environment, which enable development (Leroy & Frongillo, 2019). Consequently, linear growth retardation can be a useful marker of MAM children at risk of not meeting their development potential.

As far as we know, only one previous study has investigated the relationship between body composition and cognitive development. This was a longitudinal study among healthy Ethiopian children, which supports a beneficial effect of FFM as the authors found that more FFM at birth and a higher rate of postnatal FFM accretion predicted better development at 1 and 2 years of age, whereas FM was not associated (Abera et al., 2017; Abera, Tesfaye, Admassu, et al., 2018). However, the study found that children with higher FM at birth had more emotional and behavioural problems at 5 years (Abera et al., 2018). FM and FFM play different roles in early life growth. FM is important for brain growth and myelination and acts as an energy reserve during weaning and infection (Kuzawa, 1998). However, due to the high water content of the brain (85% at birth; Rutherford, 2001), brain growth during infancy is mainly reflected as increased FFM. This might explain the negative role of FMI we found among the children with MAM in Burkina Faso. In our study population, mean FM was low at 1.13 kg compared with 2.5 kg in a mixed feeding group of children of similar age in the United Kingdom (Wells, Davies, Fewtrell, & Cole, 2019). On the basis of these cross‐sectional data, we cannot know to what extent the body composition of the children mainly reflected differences in tissue lost during acute malnutrition or the initial body composition before malnutrition developed. There is a need for longitudinal data to investigate this further. Nevertheless, our findings highlight the relevance for programmes to focus not just on nutritional recovery of children but also on whether their diet enables them to put on FFM.

In addition, we found that PUFA status, especially n‐3 LC‐PUFAs, were associated with better development scores. As we have previously reported, family diets in our study population were predominantly cereal‐based with low consumption of animal‐source foods including cow's milk, fish, vegetable oils, and other sources of n‐3 PUFA (Yaméogo et al., 2017). Consequently, overall PUFA deficiency was not found to be a problem in this population, but levels of n‐3 LC‐PUFA were low and potentially a limiting factor for the development of children. In particular, the importance of adequate DHA for both motor and language development was seen as the coefficients sizes for DHA were large, comparable with those seen for anthropometry.

The association between malnutrition and developmental delay may reflect both actual development deficit and that malnourished, anaemic, and sick children simply lack energy or concentration to complete assessment tasks. The evaluations of participation during MDAT assessment allowed us to explore this further. We found that although the level of cooperativeness did not affect the associations with nutritional correlates, it did attenuate the associations between morbidity and MDAT *z*‐scores, suggesting that the association of morbidity with development is largely through the child's performance on the day. In contrast, although we found that the level of encouragement given by the caregiver did affect the result of MDAT assessment, it did not influence any of the identified associations between correlates and MDAT scores.

Malnutrition coexists with other factors that affect child development. Our finding that children living in large households had poorer development may be related to limited caregiver attention and other resources available for stimulation of the child. Caregiver interaction and stimulation are likely mediators of associations between anthropometry and child development, if children with better nutritional status receive more support in general. The idea that malnutrition mainly affects a child's development through its interactions with the environment has been described as the “functional isolation hypothesis” (Brown & Pollitt, 1996; Levitsky & Strupp, 1995). According to this, children who are malnourished, often ill, or small for their age may receive less stimulation from caregivers, and they may be less active in exploring their environment, both of which have a negative impact on their development. We were not able to explore this further in our study, because we lacked data on stimulation of the children.

In conclusion, this study provides new knowledge about child development and its nutrition and health‐related correlates among children with MAM. A range of nutritional markers proved relevant, indicating the importance of different periods of exposure: HAZ is a marker of long‐term nutritional supply from fetal life onwards, FFM is more sensitive to post‐natal tissue accretion, and DHA is a marker of current circulating substrate. Because many of the correlates are modifiable factors, the findings are of relevance to policymakers and planners of interventions to mitigate the detrimental effects of acute malnutrition on the long‐term developmental outcomes of children. Future studies should include longitudinal data and information on caregiver interactions and stimulation in the household to benefit further understanding of the mechanisms that link acute malnutrition to impaired child development.

## CONFLICTS OF INTEREST

The authors declare they have no conflicts of interests.

## CONTRIBUTIONS

MFO, A‐S I‐B, SF, MG, and HF conceptualised the study. A‐S I‐B piloted and adapted the tools for assessment of child development. A‐S I‐B, AO, CWY, BC, and CF collected data. MFO analysed data and wrote the first draft of the manuscript. CR, LL, HF, and MG contributed to the data analysis. All authors provided input to manuscript revisions and approved the final manuscript

## Supporting information

Table S1: Fatty acid correlates of MDAT z‐scores in 1.572 children with moderate acute malnutrition
Table S2a: MDAT z‐scores and suspected delay: Sensitivity analysis using minimum values for data imputation
Table S2b: MDAT z‐scores and suspected delay: Sensitivity analysis using maximum values for data imputation
Table S3a: Socio‐demographic and anthropometric correlates of MDAT z‐scores: Sensitivity analysis using minimum values for data imputation
Table S3b: Socio‐demographic and anthropometric correlates of MDAT z‐scores: Sensitivity analysis using maximum values for data imputation
Table S4a: Biochemical and clinical correlates of MDAT z‐scores: Sensitivity analysis using minimum values for data imputation
Table S4b: Biochemical and clinical correlates of MDAT z‐scores: Sensitivity analysis using maximum values for data imputation
