# Supplementary material for: Early development in children with moderate acute malnutrition: A cross‐sectional study in Burkina Faso
Source: Matern Child Nutr. 2019 Dec 11;16(2):e12928. doi: 10.1111/mcn.12928 (PMC7083399; doi:10.1111/mcn.12928)
Supplement: Supplementary file 1 — Table S1: Fatty acid correlates of MDAT z‐scores in 1.572 children with moderate acute malnutrition Table S2a: MDAT z‐scores and suspected delay: Sensitivity analysis using minimum values for data imputation Table S2b: MDAT z‐scores and suspected delay: Sensitivity analysis using maximum values for data imputation Table S3a: Socio‐demographic and anthropometric correlates of MDAT z‐scores: Sensitivity analysis using minimum values for data imputation Table S3b: Socio‐demographic and anthropometric correlates of MDAT z‐scores: Sensitivity analysis using maximum values for data imputation Table S4a: Biochemical and clinical correlates of MDAT z‐scores: Sensitivity analysis using minimum values for data imputation Table S4b: Biochemical and clinical correlates of MDAT z‐scores: Sensitivity analysis using maximum values for data imputation [file MCN-16-e12928-s001.doc]

Table S1: Fatty acid correlates of MDAT z-scores in 1.572 children with moderate acute malnutrition

|  |  | **Fatty acid %** | **Gross motor domain** | | **Fine motor domain** | | **Language domain** | |
| --- | --- | --- | --- | --- | --- | --- | --- | --- |
|  |  | **Mean ±SD** | **β (95% CI)** | **p** | **β (95% CI)** | **P** | **β (95% CI)** | **p** |
| Saturated fatty acids |  | 45.29 ±2.99 | -0.01 (-0.03; 0.01) | 0.52 | -0.005 (-0.02; 0.01) | 0.64 | -0.01 (-0.03; 0.01) | 0.26 |
| Monounsaturated fatty acids |  | 22.57 ±3.39 | -0.04 (-0.06; -0.02) | <0.001 | -0.02 (-0.04; -0.01) | 0.004 | -0.01 (-0.03; 0.001) | 0.06 |
| Polyunsaturated fatty acids |  | 28.53 ±2.78 | 0.05 (0.03; 0.07) | <0.001 | 0.03 (0.01; 0.05) | 0.001 | 0.02 (0.002; 0.04) | 0.03 |
| n-3 polyunsaturated fatty acids |  | 2.48 ±0.66 | 0.22 (0.14; 0.30) | <0.001 | 0.08 (-0.003; 0.16) | 0.06 | 0.12 (0.05; 0.19) | 0.001 |
| α-linolenic acid (ALA) | C18:3n-3  Boys (n=710)  Girls (n=862) | 0.21 ±0.09  0.21 ±0.09  0.22 ±0.09 | Interaction: p=0.02^a^  -1.89 (-2.71; -1.06)  -0.50 (-1.28; 0.29) | <0.001  0.21 | -0.33 (-0.90; 0.25) | 0.27 | -0.33 (-0.85; 0.19) | 0.21 |
| Eicosapentaenoic acid (EPA) | C20:5n-3 | 0.16 ±0.11 | 1.06 (0.59; 1.54) | <0.001 | 0.54 (0.06; 1.02) | 0.03 | 0.71 (0.28; 1.14) | 0.001 |
| n-3 docosapentaenoic acid (n-3 DPA) | C22:5n-3 | 0.44 ±0.12 | 0.74 (0.32; 1.16) | 0.001 | 0.01 (-0.41; 0.44) | 0.96 | 0.35 (-0.03; 0.73) | 0.07 |
| Docosahexaenoic acid (DHA) | C22:6n-3 | 1.64 ±0.53 | 0.28 (0.18; 0.38) | <0.001 | 0.11 (0.01; 0.21) | 0.04 | 0.15 (0.06; 0.24) | 0.001 |
| n-6 polyunsaturated fatty acids |  | 26.00 ±2.66 | 0.04 (0.02; 0.06) | <0.001 | 0.03 (0.01; 0.05) | 0.003 | 0.01 (-0.004; 0.03) | 0.14 |
| Linoleic acid (LA) | C18:2n-6 | 16.23 ±2.26 | 0.01 (-0.01; 0.03) | 0.44 | 0.03 (0.01; 0.06) | 0.004 | 0.008 (-0.01; 0.03) | 0.46 |
| Dihomo-γ-linolenic acid (DGLA) | C20:3n-6 | 0.86 ±0.21 | 0.43 (0.18; 0.67) | 0.001 | 0.22 (-0.03; 0.46) | 0.09 | 0.20 (-0.02; 0.43) | 0.07 |
| Arachidonic acid (AA) | C20:4n-6 | 7.08 ±1.54 | 0.08 (0.04; 0.11) | <0.001 | 0.01 (-0.02; 0.05) | 0.44 | 0.02 (-0.01; 0.05) | 0.22 |
| Adrenic acid (AdA) | C22:4n-6 | 0.95 ±0.27 | 0.20 (0.004; 0.39) | 0.045 | -0.06 (-0.26; 0.13) | 0.52 | 0.02 (-0.16; 0.19) | 0.83 |
| n-6 docosapentaenoic acid (n-6 DPA) | C22:5n-6  Boys (n=710)  Girls (n=862) | 0.38 ±0.13  0.39 ±0.13  0.38 ±0.13 | 0.22 (-0.18; 0.63) | 0.28 | -0.32 (-0.73; 0.09) | 0.12 | Interaction: p=0.008^a^  0.47 (-0.10; 1.03)  -0.55 (-1.03; -0.07) | 0.11  0.02 |
| Mead acid | C20:3n-9 | 0.07 ±0.03 | 0.19 (-1.72; 2.10) | 0.84 | -0.09 (-2.03; 1.85) | 0.93 | 1.54 (-0.20; 3.27) | 0.08 |
| Indicator of low PUFA status |  |  |  |  |  |  |  |  |
| Mead acid:AA ratio | C20:3n-9/C20:4n-6 | 0.01 ±0.005 | -10.96 (-21.44; -0.49) | 0.04 | -1.99 (-12.61; 8.63) | 0.71 | 3.88 (-5.66; 13.41) | 0.43 |
| Indicators of low n-3 PUFA status |  |  |  |  |  |  |  |  |
| n-6 DPA:DHA ratio | C22:5n-6/C22:6n-3  Boys (n=710)  Girls (n=862) | 0.25 ±0.10 | -0.84 (-1.37; -0.32) | 0.002 | Interaction: p=0.04^b^  -0.25 (-1.02; 0.52)  -1.32 (-2.07; -0.58) | 0.53  <0.001 | -0.62 (-1.10; -0.14) | 0.01 |
| n-6 PUFA:n-3 PUFA ratio |  | 11.13 ±2.86 | -0.04 (-0.05; -0.02) | <0.001 | -0.01 (-0.03; 0.01) | 0.47 | -0.02 (-0.04; -0.01) | 0.006 |

Data are mean difference (95% CI) from linear mixed models adjusted for age, sex, month of inclusion, and site (random effects). Fatty acid levels are given in weight percent relative to total fatty acid concentration (FA%). Mean total acid concentration was 417 (±183) μg/100 μL whole blood. ^a^ Due to interaction, sex-specific estimates are given.

**Table S2a: MDAT z-scores and suspected delay: Sensitivity analysis using minimum values for data imputation**

| **MDAT domain** | **Mean z-score (95% CI)** | **Developmental delay, n (%)** |
| --- | --- | --- |
| Gross motor | -0.39 (-0.45; -0.34) | 186 (11.6) |
| Fine motor | 0.54 (0.48; 0.59) | 74 (4.6) |
| Language | -0.91 (-0.96; -0.86) | 390 (24.3) |

MDAT = adapted version of the Malawi Development Assessment Tool. Z-scores are based on MDAT reference population data (Gladstone 2010). Domain z–scores below -1.64 are suspect for developmental delay.

**Table S2b: MDAT z-scores and suspected delay: Sensitivity analysis using maximum values for data imputation**

| **MDAT domain** | **Mean z-score (95% CI)** | **Developmental delay, n (%)** |
| --- | --- | --- |
| Gross motor | -0.39 (-0.45; -0.34) | 186 (11.6) |
| Fine motor | 0.59 (0.53; 0.65) | 74 (4.6) |
| Language | -0.85 (-0.91; -0.79) | 380 (23.6) |

MDAT = adapted version of the Malawi Development Assessment Tool. Z-scores are based on MDAT reference population data (Gladstone 2010). Domain z–scores below -1.64 are suspect for developmental delay.

**Table S3a: Socio-demographic and anthropometric correlates of MDAT z-scores: Sensitivity analysis** using minimum values for data imputation

|  |  | **Gross motor domain** | | **Fine motor domain** | | **Language domain** | |
| --- | --- | --- | --- | --- | --- | --- | --- |
|  | **n** | **β (95% CI)** | **P** | **β (95% CI)** | **p** | **β (95% CI)** | **p** |
| **Socio-demographic characteristics** |  |  |  |  |  |  |  |
| Sex |  |  |  |  |  |  |  |
| Boys | 729 | Ref |  | Ref |  | Ref |  |
| Girls | 879 | -0.06 (-0.16; 0.04) | 0.22 | -0.04 (-0.14; 0.06) | 0.43 | 0.03 (-0.06; 0.12) | 0.53 |
| Maternal education level |  |  |  |  |  |  |  |
| None | 1,373 | Ref | 0.63 | Ref | 0.90 | Ref | 0.87 |
| Primary school incomplete | 156 | 0.09 (-0.09; 0.28) |  | 0.02 (-0.17; 0.21) |  | -0.03 (-0.20; 0.14) |  |
| Primary school complete or higher | 73 | 0.01 (-0.19; 0.22) |  | 0.04 (-0.17; 0.25) |  | 0.04 (-0.15; 0.22) |  |
| Maternal marital status |  |  |  |  |  |  |  |
| Married, monogamous | 645 | Ref |  | Ref |  | Ref |  |
| Married, polygamous | 696 | -0.10 (-0.21; 0.01) | 0.07 | -0.03 (-0.14; 0.08) | 0.56 | -0.07 (-0.17; 0.03) | 0.17 |
| Unmarried | 256 | -0.20 (-0.35; -0.05) | 0.01 | -0.05 (-0.21; 0.10) | 0.49 | -0.01 (-0.15; 0.13) | 0.89 |
| Household size |  |  |  |  |  |  |  |
| ≤ 6 household members | 263 | Ref | 0.01 | Ref | 0.10 | Ref | 0.02 |
| 7-12 household members | 713 | -0.02 (-0.16; 0.13) |  | -0.02 (-0.16; 0.13) |  | -0.04 (-0.17; 0.09) |  |
| ≥ 13 household members | 632 | -0.17 (-0.32; -0.02) |  | -0.13 (-0.28; 0.02) |  | -0.17 (-0.30; -0.03) |  |
| **Anthropometry** |  |  |  |  |  |  |  |
| Mid-upper arm circumference (MUAC), mm^a^ | 1,271 | 0.03 (0.01; 0.06) | 0.002 | 0.03 (0.01; 0.05) | 0.01 | -0.004 (-0.02; 0.02) | 0.67 |
| Weight-for-height z-score (WHZ)^b^ | 1,141 | 0.08 (-0.13; 0.29) | 0.48 | 0.37 (0.17; 0.58) | <0.001 | 0.16 (-0.03; 0.36) | 0.10 |
| Height-for-age z-score (HAZ) | 1,608  Boys: 729  Girls: 879 | 0.29 (0.24; 0.33) | <0.001 | 0.29 (0.24; 0.33) | <0.001 | Interaction: p=0.002^c^  0.26 (0.20; 0.32)  0.12 (0.06; 0.18) | <0.001  <0.001 |
| MAM-defining criteria |  |  |  |  |  |  |  |
| MUAC only | 467 | Ref |  | Ref |  | Ref |  |
| WHZ only | 337 | 0.31 (0.15; 0.47) | <0.001 | 0.24 (0.08; 0.40) | 0.004 | 0.16 (0.02; 0.30) | 0.03 |
| MUAC and WHZ | 804 | 0.06 (-0.06; 0.18) | 0.32 | 0.10 (-0.02; 0.22) | 0.11 | 0.05 (-0.06; 0.16) | 0.37 |
| WHZ only, adjusted for HAZ | 337 | 0.06 (-0.09; 0.22) | 0.41 | -0.01 (-0.17; 0.15) | 0.89 | -0.01 (-0.15; 0.14) | 0.91 |
| MUAC and WHZ, adjusted for HAZ | 804 | -0.04 (-0.16; 0.08) | 0.49 | -0.004 (-0.12; 0.11) | 0.94 | -0.02 (-0.13; 0.09) | 0.71 |
| **Body composition** |  |  |  |  |  |  |  |
| Fat-free mass, kg | 1,489 | 0.48 (0.39; 0.58) | <0.001 | 0.38 (0.29; 0.48) | <0.001 | 0.30 (0.21; 0.39) | <0.001 |
| Fat mass, kg | 1,489 | -0.03 (-0.17; 0.12) | 0.72 | 0.14 (-0.003; 0.28) | 0.056 | -0.04 (-0.17; 0.09) | 0.55 |
| Fat-free mass index, kg/m^2^ | 1,489 | 0.02 (-0.05; 0.08) | 0.58 | -0.03 (-0.09; 0.04) | 0.39 | 0.01 (-0.05; 0.07) | 0.73 |
| Fat mass index, kg/m^2^ | 1,489 | -0.13 (-0.20; -0.05) | 0.001 | -0.03 (-0.11; 0.04) | 0.37 | -0.07 (-0.14; -0.05) | 0.036 |
| Fat-free mass index, kg/m^2^, adjusted for HAZ | 1,489 | 0.10 (0.04; 0.16) | 0.002 | 0.05 (-0.01; 0.11) | 0.12 | 0.07 (0.01; 0.12) | 0.03 |
| Fat mass index, kg/m^2^, adjusted for HAZ | 1,489 | -0.11 (-0.18; -0.04) | 0.002 | -0.02 (-0.09; 0.05) | 0.61 | -0.06 (-0.13; 0.005) | 0.07 |

Data are mean difference (95% CI) from linear mixed models adjusted for age, sex, month of inclusion, and site (random effects). ^a^ Includes children with MUAC <125 mm only. ^b^ Includes children with WHZ <-2 only. ^c^ Due to interaction, sex-specific estimates are given.

**Table S3b: Socio-demographic and anthropometric correlates of MDAT z-scores: Sensitivity analysis** using maximum values for data imputation

|  |  | **Gross motor domain** | | **Fine motor domain** | | **Language domain** | |
| --- | --- | --- | --- | --- | --- | --- | --- |
|  | **n** | **β (95% CI)** | **P** | **β (95% CI)** | **p** | **β (95% CI)** | **p** |
| **Socio-demographic characteristics** |  |  |  |  |  |  |  |
| Sex |  |  |  |  |  |  |  |
| Boys | 729 | Ref |  | Ref |  | Ref |  |
| Girls | 879 | -0.06 (-0.16; 0.04) | 0.21 | -0.04 (-0.14; 0.07) | 0.47 | 0.03 (-0.07; 0.14) | 0.53 |
| Maternal education level |  |  |  |  |  |  |  |
| None | 1,373 | Ref | 0.64 | Ref | 0.87 | Ref | 0.76 |
| Primary school incomplete | 156 | 0.09 (-0.10; 0.28) |  | 0.01 (-0.19; 0.21) |  | -0.06 (-0.25; 0.13) |  |
| Primary school complete or higher | 73 | 0.01 (-0.20; 0.22) |  | 0.06 (-0.16; 0.28) |  | 0.04 (-0.17; 0.26) |  |
| Maternal marital status |  |  |  |  |  |  |  |
| Married, monogamous | 645 | Ref |  | Ref |  | Ref |  |
| Married, polygamous | 696 | -0.10 (-0.21; 0.01) | 0.07 | -0.06 (-0.18; 0.06) | 0.32 | -0.10 (-0.21; 0.02) | 0.09 |
| Unmarried | 256 | -0.21 (-0.36; -0.05) | 0.01 | -0.10 (-0.26; 0.07) | 0.25 | 0.01 (-0.15; 0.17) | 0.90 |
| Household size |  |  |  |  |  |  |  |
| ≤ 6 household members | 263 | Ref | 0.01 | Ref | 0.04 | Ref | 0.01 |
| 7-12 household members | 713 | -0.02 (-0.17; 0.12) |  | -0.05 (-0.21; 0.11) |  | -0.06 (-0.21; 0.09) |  |
| ≥ 13 household members | 632 | -0.18 (-0.33; -0.03) |  | -0.18 (-0.35; -0.02) |  | -0.22 (-0.37; -0.06) |  |
| **Anthropometry** |  |  |  |  |  |  |  |
| Mid-upper arm circumference (MUAC), mm^a^ | 1,271 | 0.03 (0.01; 0.05) | 0.002 | 0.03 (0.01; 0.06) | 0.01 | -0.01 (-0.03; 0.01) | 0.45 |
| Weight-for-height z-score (WHZ)^b^ | 1,141 | 0.07 (-0.14; 0.28) | 0.51 | 0.45 (0.23; 0.68) | <0.001 | 0.16 (-0.06; 0.38) | 0.16 |
| Height-for-age z-score (HAZ) | 1,608  Boys: 729  Girls: 879 | 0.29 (0.24; 0.33) | <0.001 | 0.31 (0.26; 0.35) | <0.001 | Interaction: p=<0.001^c^  0.31 (0.24; 0.38)  0.12 (0.06; 0.19) | <0.001  <0.001 |
| MAM-defining criteria |  |  |  |  |  |  |  |
| MUAC only | 467 | Ref |  | Ref |  | Ref |  |
| WHZ only | 337 | 0.31 (0.15; 0.47) | <0.001 | 0.24 (0.07; 0.42) | 0.005 | 0.16 (-0.01; 0.32) | 0.06 |
| MUAC and WHZ | 804 | 0.06 (-0.06; 0.18) | 0.33 | 0.09 (-0.04; 0.22) | 0.16 | 0.04 (-0.08; 0.17) | 0.52 |
| WHZ only, adjusted for HAZ | 337 | 0.07 (-0.09; 0.22) | 0.41 | -0.02 (-0.19; 0.15) | 0.81 | -0.04 (-0.20; 0.13) | 0.67 |
| MUAC and WHZ, adjusted for HAZ | 804 | -0.04 (-0.16; 0.07) | 0.47 | -0.02 (-0.14; 0.11) | 0.79 | -0.04 (-0.16; 0.08) | 0.53 |
| **Body composition** |  |  |  |  |  |  |  |
| Fat-free mass, kg | 1,489 | 0.48 (0.39; 0.58) | <0.001 | 0.43 (0.32; 0.53) | <0.001 | 0.35 (0.25; 0.45) | <0.001 |
| Fat mass, kg | 1,489 | -0.02 (-0.17; 0.12) | 0.74 | 0.14 (-0.02; 0.29) | 0.08 | -0.07 (-0.22; 0.08) | 0.36 |
| Fat-free mass index, kg/m^2^ | 1,489 | 0.02 (-0.05; 0.08) | 0.61 | -0.02 (-0.09; 0.05) | 0.55 | 0.02 (-0.05; 0.08) | 0.64 |
| Fat mass index, kg/m^2^ | 1,489 | -0.13 (-0.20; -0.05) | 0.001 | -0.04 (-0.12; 0.04) | 0.34 | -0.09 (-0.17; -0.01) | 0.023 |
| Fat-free mass index, kg/m^2^, adjusted for HAZ | 1,489 | 0.10 (0.03; 0.16) | 0.002 | 0.06 (-0.004; 0.13) | 0.07 | 0.08 (0.01; 0.15) | 0.02 |
| Fat mass index, kg/m^2^, adjusted for HAZ | 1,489 | -0.11 (-0.18; -0.04) | 0.002 | -0.02 (-0.10; 0.05) | 0.58 | -0.08 (-0.15; -0.002) | 0.045 |

Data are mean difference (95% CI) from linear mixed models adjusted for age, sex, month of inclusion, and site (random effects). ^a^ Includes children with MUAC <125 mm only. ^b^ Includes children with WHZ <-2 only. ^c^ Due to interaction, sex-specific estimates are given.

**Table S4a: Biochemical and clinical correlates of MDAT z-scores: Sensitivity analysis** using minimum values for data imputation

|  |  | **Gross motor domain** | | **Fine motor domain** | | **Language domain** | |
| --- | --- | --- | --- | --- | --- | --- | --- |
|  | **n** | **β (95% CI)** | **P** | **β (95% CI)** | **p** | **β (95% CI)** | **p** |
| **Long chain polyunsaturated fatty acids, %FA**^a^ |  |  |  |  |  |  |  |
| Docosahexaenoic acid (DHA) | 1,572 | 0.28 (0.18; 0.38) | <0.001 | 0.11 (0.01; 0.21) | 0.04 | 0.15 (0.06; 0.24) | 0.001 |
| Arachidonic acid (AA) | 1,572 | 0.08 (0.04; 0.11) | <0.001 | 0.01 (-0.02; 0.05) | 0.44 | 0.02 (-0.01; 0.05) | 0.22 |
| Indicator of low PUFA status |  |  |  |  |  |  |  |
| Mead acid: AA ratio | 1,572 | -10.96 (-21.44; -0.49) | 0.04 | -1.99 (-12.61; 8.63) | 0.71 | 3.88 (-5.66; 13.41) | 0.43 |
| Indicator of low n-3 PUFA status |  |  |  |  |  |  |  |
| n-6 docosapentaenoic acid (n-6 DPA): DHA ratio | 1,572  Boys: 710  Girls: 862 | -0.84 (-1.37; -0.32) | 0.002 | Interaction: p=0.04^b^  -0.25 (-1.02; 0.52)  -1.32 (-2.07; -0.58) | 0.53  <0.001 | -0.62 (-1.10; -0.14) | 0.01 |
| **Haemoglobin and iron** |  |  |  |  |  |  |  |
| Hb, g/dL | 1,608 | 0.11 (0.07; 0.14) | <0.001 | 0.08 (0.04; 0.11) | <0.001 | 0.06 (0.03; 0.09) | <0.001 |
| Serum ferritin, inflammation-corrected^c^, ln(µg/L) | 1,555  Boys: 700  Girls: 855 | -0.01 (-0.06; 0.05) | 0.86 | 0.001 (-0.06; 0.06) | 0.97 | Interaction: p=0.044^b^  0.08 (0.004; 0.16)  -0.02 (-0.09; 0.04) | 0.04  0.49 |
| ≥ 12 µg/L | 595 | Ref |  | Ref |  | Ref |  |
| < 12 µg/L | 960 | -0.004 (-0.11; 0.10) | 0.94 | 0.04 (-0.07; 0.15) | 0.45 | -0.04 (-0.14; 0.06) | 0.42 |
| Soluble transferrin receptors, ln(mg/L) | 1,564 | -0.36 (-0.48; -0.24) | <0.001 | -0.17 (-0.30; -0.05) | 0.006 | -0.21 (-0.32; -0.10) | <0.001 |
| ≤ 8.3 mg/L | 268 | Ref | <0.001 | Ref | 0.03 | Ref | 0.02 |
| >8.3 – <15 mg/L | 742 | -0.08 (-0.22; 0.07) |  | -0.001 (-0.15; 0.14) |  | 0.003 (-0.13; 0.13) |  |
| ≥ 15 mg/L | 554 | -0.34 (-0.50; -0.19) |  | -0.15 (-0.31; 0.01) |  | -0.14 (-0.28; 0.002) |  |
| Anaemia |  |  |  |  |  |  |  |
| No anaemia | 468 | Ref |  | Ref |  | Ref |  |
| Anaemia with iron deficiency ^d^ | 469 | -0.19 (-0.32; -0.05) | 0.006 | -0.15 (-0.28; -0.01) | 0.04 | -0.13 (-0.25; -0.01) | 0.04 |
| Anaemia without iron deficiency ^e^ | 618 | -0.16 (-0.28; -0.03) | 0.02 | -0.19 (-0.32; -0.06) | 0.004 | -0.11 (-0.23; 0.001) | 0.052 |
| **Morbidity** |  |  |  |  |  |  |  |
| Illness within the last two weeks |  |  |  |  |  |  |  |
| No | 991 | Ref |  | Ref |  | Ref |  |
| Yes | 608 | -0.17 (-0.28; -0.06) | 0.003 | -0.20 (-0.31; -0.09) | <0.001 | -0.13 (-0.23; -0.03) | 0.01 |
| Malaria (positive test) |  |  |  |  |  |  |  |
| Negative test | 956 | Ref |  | Ref |  | Ref |  |
| Positive test | 644 | -0.14 (-0.25; -0.02) | 0.02 | -0.10 (-0.22; 0.02) | 0.10 | -0.06 (-0.17; 0.04) | 0.25 |
| Serum CRP, ln(mg/L) | 1,555 | -0.06 (-0.09; -0.03) | <0.001 | -0.08 (-0.11; -0.05) | <0.001 | -0.07 (-0.09; -0.04) | <0.001 |
| <5 mg/L | 1,002 | Ref | <0.001 | Ref | <0.001 | Ref | <0.001 |
| ≥5 to <10 mg/L | 183 | -0.19 (-0.35; -0.03) |  | -0.10 (-0.26; 0.06) |  | -0.11 (-0.26; 0.04) |  |
| ≥10 mg/L | 379 | -0.21 (-0.33; -0.09) |  | -0.29 (-0.42; -0.17) |  | -0.25 (-0.37; -0.14) |  |

Data are mean differences (95% CI) from linear mixed models adjusted for age, sex, month of inclusion, and site (random effects). ^a^ LCPUFA data are given in weight percent relative to total fatty acid concentration (FA%). ^b^ Due to interaction, sex-specific estimates are given. ^c^ Corrected in linear model with C-reactive protein (CRP), α_1_-acid glycoprotein (AGP) and morbidity covariates (malaria, lower respiratory tract infections and history of fever). ^d^ Defined as haemoglobin <11 g/dL and SFAI <12 μg/L. ^e^ Defined as haemoglobin < 11 g/dL and SFAI ≥ 12 μg/L.

**Table S4b: Biochemical and clinical correlates of MDAT z-scores: Sensitivity analysis** using maximum values for data imputation

|  |  | **Gross motor domain** | | **Fine motor domain** | | **Language domain** | |
| --- | --- | --- | --- | --- | --- | --- | --- |
|  | **n** | **β (95% CI)** | **P** | **β (95% CI)** | **p** | **β (95% CI)** | **p** |
| **Long chain polyunsaturated fatty acids, %FA**^a^ |  |  |  |  |  |  |  |
| Docosahexaenoic acid (DHA) | 1,572 | 0.28 (0.18; 0.38) | <0.001 | 0.12 (0.01; 0.23) | 0.03 | 0.18 (0.08; 0.29) | 0.001 |
| Arachidonic acid (AA) | 1,572 | 0.07 (0.04; 0.11) | <0.001 | 0.01 (-0.02; 0.05) | 0.46 | 0.02 (-0.02; 0.05) | 0.30 |
| Indicator of low PUFA status |  |  |  |  |  |  |  |
| Mead acid: AA ratio | 1,572 | -10.48 (-20.44; 0.01) | 0.050 | -0.56 (-11.91; 10.80) | 0.92 | 2.93 (-8.03; 13.89) | 0.60 |
| Indicator of low n-3 PUFA status |  |  |  |  |  |  |  |
| n-6 docosapentaenoic acid (n-6 DPA): DHA ratio | 1,572  Boys: 710  Girls: 862 | -0.84 (-1.36; -0.31) | 0.002 | Interaction: p=0.03^b^  -0.36 (-1.20; 0.47)  -1.39 (-2.12; -0.65) | 0.40  <0.001 | -0.89 (-1.44; -0.34) | 0.002 |
| **Haemoglobin and iron** |  |  |  |  |  |  |  |
| Hb, g/dL | 1,608 | 0.11 (0.07; 0.14) | <0.001 | 0.08 (0.05; 0.12) | <0.001 | 0.07 (0.04; 0.11) | <0.001 |
| Serum ferritin, inflammation-corrected^c^, ln(µg/L) | 1,555  Boys: 700  Girls: 855 | -0.003 (-0.06; 0.05) | 0.91 | 0.02 (-0.04; 0.08) | 0.47 | Interaction: p=0.02^b^  0.12 (0.03; 0.21)  -0.02 (-0.10; 0.05) | 0.01  0.52 |
| ≥ 12 µg/L | 595 | Ref |  | Ref |  | Ref |  |
| < 12 µg/L | 960 | -0.006 (-0.11; 0.10) | 0.91 | 0.004 (-0.11; 0.12) | 0.95 | -0.08 (-0.19; 0.03) | 0.17 |
| Soluble transferrin receptors, ln(mg/L) | 1,564 | -0.36 (-0.48; -0.24) | <0.001 | -0.21 (-0.34; -0.07) | 0.002 | -0.27 (-0.39; -0.14) | <0.001 |
| ≤ 8.3 mg/L | 268 | Ref | <0.001 | Ref | 0.02 | Ref | 0.005 |
| >8.3 – <15 mg/L | 742 | -0.07 (-0.22; 0.07) |  | -0.0001 (-0.16; 0.16) |  | 0.01 (-0.14; 0.16) |  |
| ≥ 15 mg/L | 554 | -0.34 (-0.50; -0.19) |  | -0.17 (-0.34; -0.003) |  | -0.19 (-0.35; -0.03) |  |
| Anaemia |  |  |  |  |  |  |  |
| No anaemia | 468 | Ref |  | Ref |  | Ref |  |
| Anaemia with iron deficiency ^d^ | 469 | -0.19 (-0.32; -0.05) | 0.006 | -0.19 (-0.34; -0.04) | 0.01 | -0.18 (-0.32; -0.03) | 0.02 |
| Anaemia without iron deficiency ^e^ | 618 | -0.16 (-0.28; -0.03) | 0.02 | -0.20 (-0.34; -0.06) | 0.004 | -0.13 (-0.26; 0.01) | 0.06 |
| **Morbidity** |  |  |  |  |  |  |  |
| Illness within the last two weeks |  |  |  |  |  |  |  |
| No | 991 | Ref |  | Ref |  | Ref |  |
| Yes | 608 | -0.17 (-0.27; -0.06) | 0.003 | -0.21 (-0.32; -0.09) | 0.001 | -0.10 (-0.22; 0.01) | 0.07 |
| Malaria (positive test) |  |  |  |  |  |  |  |
| Negative test | 956 | Ref |  | Ref |  | Ref |  |
| Positive test | 644 | -0.14 (-0.25; -0.02) | 0.02 | -0.10 (-0.23; 0.02) | 0.10 | -0.08 (-0.20; 0.04) | 0.18 |
| Serum CRP, ln(mg/L) | 1,555 | -0.06 (-0.09; -0.03) | <0.001 | -0.08 (-0.11; -0.05) | <0.001 | -0.07 (-0.10; -0.04) | <0.001 |
| <5 mg/L | 1,002 | Ref | 0.001 | Ref | <0.001 | Ref | <0.001 |
| ≥5 to <10 mg/L | 183 | -0.19 (-0.35; -0.03) |  | -0.11 (-0.28; 0.07) |  | -0.16 (-0.33; 0.01) |  |
| ≥10 mg/L | 379 | -0.21 (-0.33; -0.08) |  | -0.32 (-0.45; -0.19) |  | -0.27 (-0.40; -0.14) |  |

Data are mean differences (95% CI) from linear mixed models adjusted for age, sex, month of inclusion, and site (random effects). ^a^ LCPUFA data are given in weight percent relative to total fatty acid concentration (FA%). ^b^ Due to interaction, sex-specific estimates are given. ^c^ Corrected in linear model with C-reactive protein (CRP), α_1_-acid glycoprotein (AGP) and morbidity covariates (malaria, lower respiratory tract infections and history of fever). ^d^ Defined as haemoglobin <11 g/dL and SFAI <12 μg/L. ^e^ Defined as haemoglobin < 11 g/dL and SFAI ≥ 12 μg/L.
